# Arthritis Caused by MRSA CC398 in a Patient without Animal Contact, Japan

**DOI:** 10.3201/eid2612.203738

**Published:** 2020-12

**Authors:** Hidemasa Nakaminami

**Affiliations:** Tokyo University of Pharmacy and Life Sciences, Tokyo, Japan

**Keywords:** livestock-associated methicillin-resistant *Staphylococcus aureus*, CC398, ST1232, Panton-Valentine leukocidin, immune evasion cluster, MRSA, CC398, clonal complex, bacteria, Japan, sequence type, staphylococci, antimicrobial resistance

**In Response:** In our article (*1*), we hypothesized that the transmission route of the Panton-Valentine leukocidin (PVL)–positive sequence type (ST) 1232 (CC398) MRSA strain is not only from humans but also from imported edible meat for humans. However, in their letter, Larsen and Larsen (*2*) indicated that *S. aureus* CC398 includes 2 major MRSA variants with distinct genetic and epidemiologic properties; 1 being a highly transmissible and virulent human variant comprising both PVL-positive and PVL-negative strains, and the other being a more benign PVL-negative livestock-associated variant (*3*). The presence of PVL genes and immune evasion cluster (IEC) genes in CC398 strain provides supportive evidence for the association of human colonization or infections. Furthermore, they showed that most case-patients in Denmark who were colonized or infected with PVL-positive MRSA CC398 strains of the human variant have links to countries in mainland Asia (*4*).

Actually, we confirmed *scn*, *chp*, and *sak* of the IEC genes in the PVL-positive ST1232 strain. Hence, as Larsen and Larsen suggested, the ST1232 strain might be a human variant of CC398. We recently reported a second case of the ST1232 strain with characteristics similar to the previous patient in Japan (*5*). The data strongly suggest that the incidence of human variant of CC398 has been increasing in Japan. Therefore, I agree with their opinion that accurate discrimination of the human variant of MRSA CC398 from the livestock-associated variant is essential for maintaining effective MRSA infection control. I presume that detection of PVL and IEC genes might be a useful simplified marker for classification of the human variant of CC398.
